# Using the pathogen-host interactions database (PHI-base) to investigate plant pathogen genomes and genes implicated in virulence

**DOI:** 10.3389/fpls.2015.00605

**Published:** 2015-08-06

**Authors:** Martin Urban, Alistair G. Irvine, Alayne Cuzick, Kim E. Hammond-Kosack

**Affiliations:** Department of Plant Biology and Crop Science, Rothamsted ResearchHarpenden, UK

**Keywords:** gene regulatory networks, plant diseases, protein interaction mapping, genetic recombination, comparative genomics, horizontal gene transfer, phytopathogens, emerging diseases

## Abstract

New pathogen-host interaction mechanisms can be revealed by integrating mutant phenotype data with genetic information. PHI-base is a multi-species manually curated database combining peer-reviewed published phenotype data from plant and animal pathogens and gene/protein information in a single database.

PHI-base is a multi-species knowledge database capturing the phenotypes available on forward and reverse mutants from 231 pathogenic organisms described in the literature. Plant pathogens represent 60% of the species within PHI-base. Simple and advanced search tools, available at www.phi-base.org, allow users to query PHI-base directly. Flat file downloads enable larger comparative biology studies, systems biology approaches and a richer annotation of genomes, transcriptomes and proteome data sets. Since 2014, phenotype information from PHI-base is directly displayed in pathogen genome browsers accessible at www.phytopathdb.org (Kersey et al., 2014). PHI-base regularly interacts with the international community to provide researchers with effective query tools and new data types to study pathogen-host interactions.

Available online since 2005, PHI-base catalogs experimentally verified pathogenicity, virulence and effector genes from fungal, protist, and bacterial pathogens which infect animal, plant, fish, insect, and/or fungal hosts (Urban et al., 2015). PHI-base is a database devoted to the identification and presentation of information on pathogenicity and effector genes and their host interactions. PHI-base was developed out of a need for a knowledge database enabling the discovery of candidate targets in medically and agronomically important species for intervention with chemistries and/or host modifications. Recent bioinformatics studies enabled by whole-database downloads of PHI-base, include comparative analyses, genome/transcript and proteome annotations, and system biology approaches (Hu et al., 2014; Zhang et al., 2014). PHI-base has been cited in 122 published articles including genetics, genomics and bioinformatics research and review articles (for an up-to-date list, see the “About” page of the PHI-base website). In 2014, the web site had more than 6000 visits and the entire content was downloaded >300 times. Phenotypic outcome data from PHI-base are also displayed directly in genome browsers as permanent tracks in public genome sequence resources such as Ensembl Fungi (Figure 1). Through a simple system of color coding and using nine high level PHI-base phenotypes (Urban et al., 2015), genomic features such as pathogenicity islands can directly be investigated.

**Figure 1:**
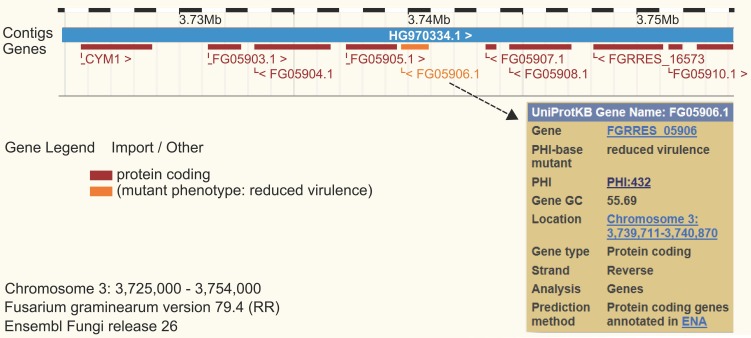
**Ensembl genome browser view for *Fusarium graminearum.*** The website at http://fungi.ensembl.org/ Fusarium_graminearum was searched for the gene id FGSG_05906 encoding the secreted lipase gene Fg*fgl1*. The PHI-base phenotype of the mutant is displayed and color coded in orange as “reduced virulence.”

The latest PHI-base release, version 3.8, contains a total of 3562 pathogen genes tested in 3697 plant- and 1257 animal-pathogen interactions. The top 10 plant pathogens are listed in Table 1. The data in PHI-base is obtained by biocuration scientists who extract the relevant information from peer-reviewed published articles in a manual curation workflow that includes the evaluation of full text, figures and tables, to create computable data records using controlled vocabularies and ontologies. This approach generates a unique level of detail and breadth compared to automated approaches and thus provides instant access to a catalog of gold standard curated gene/protein function and host phenotypic information. Various complementary multi-species databases on pathogens exist that provide gene function annotation. Each specializes in particular species/pathogen groups and/or uses only automated approaches to knowledge acquisition (Table 2). Other resources are more geared to the analysis of host-pathogen interactions by providing protein-protein interaction (PPI) data, transcriptomics and genome assembly datasets or provide WEB portal linking to multiple databases and providing advanced analysis tools.

**Table 1:** **Top 10 plant pathogen species in PHI-base**.

**Species**	**Genes curated**	**Interactions curated**
*Fusarium graminearum*^a^	966	1078
*Magnaporthe oryzae*^b^	423	662
*Ustilago maydis*	197	252
*Botrytis cinerea*^c^	86	210
*Pseudomonas syringae*	73	140
*Fusarium oxysporum*	58	85
*Hyaloperonospora arabidopsidis*^d^	55	67
*Zymoseptoria tritici*^e^	41	42
*Parastagonospora nodorum*^f^	40	46
*Leptosphaeria maculans*	17	21

Species name synonyms.

a Gibberella zeae.

b Magnaporthe grisea.

c Botryotinia fuckeliana.

d Hyaloperonospora parasitica or Peronospora parasitica.

e Mycosphaerella graminicola or Septoria tritici.

f Stagonospora nodorum, Phaeosphaeria nodorum, or Septoria nodorum.

**Table 2:** **Synopsis of complementary multi-species pathogen databases and their specialisms^a^**.

**Database**	**Content**	**URL (http://)**	**Comments**
**MULTI-SPECIES DATABASES PROVIDING GENE FUNCTION ANNOTATION**			
AgBase	12 animals, 7 plant, 26 microbial species including 15 viruses	agbase.msstate.edu	Agricultural plant and animal gene products database with a focus on GO annotation
CPGR	138 plant pathogen genomes and transcript collections	cpgr.plantbiology.msu.edu/index.html	The comprehensive phytopathogen genomics resource is focused on enabling the development of diagnostic molecular markers
DFVF	2048 genes	sysbio.unl.edu/DFVF	Covers fungal pathogen genes and virulence factors acquired using a text-mining approach
FungiDB	75 fungal genomes	fungidb.org	The fungal and oomycete genomics resources database provides graphical tools for data mining. Users have the option to search GO annotation and comments entered by users
PHIDIAS	36 species	www.phidias.us	Pathogen-host interaction data integration and analysis system with focus on human and animal priority pathogens with regard to public health
VFDB	25 species	www.mgc.ac.cn/VFs	Focus on virulence factors of human and animal bacterial pathogens
**PROTEIN-PROTEIN INTERACTION DATABASES**			
HoPaCI-DB	4272 interactions	http://mips.helmholtz-muenchen.de/HoPaCI/	Host-*Pseudomonas aeruginosa* and *Coxiella burnetti* interaction DataBase is manually curated with focus on mammalian, *Drosophila melanogaster* or *Danio rerio* generated data
HPIDB	>68 host and 567 pathogen species	http://www.agbase.msstate.edu/hpi/main.html	The host-pathogen interaction database focused on experimental protein-protein interactions from diverse mammalian and plant hosts infected by influenza, bacteria and fungi
**TRANSCRIPTOMICS DATABASES**			
PLEXdb	Vast, includes 12 fungal pathogens	www.plexdb.org	Transcriptomics database only on plants, pathogens and their interactions
Eumicrobedb	Vast	www.eumicrobedb.org/transcripts	Oomycetes transcriptomics database providing transcriptome and EST data
**GENOME BROWSERS**			
Broad-fungal genomics	>100 species	http://www.broadinstitute.org/scientific- community/science/projects/fungal-genome- initiative	Allows comparative analysis for fungal organisms including human and plant pathogens. Gene annotation searchable by keyword. Includes non-pathogenic species
Ensembl genomes	Vast number of genomes	www.ensemblgenomes.org	Non-vertebrate species genome browser suite with dedicated sub-portals for bacteria, fungi, protists, and plants species. PHI-base phenotypes directly displayed in individual genome browsers and accessible via the multiple species analysis tool BioMart
JGI-mycoCosm	Vast number of genomes	genome.jgi.doe.gov/programs/fungi	A genome portal for 100 s of pathogenic and non-pathogenic fungal species. No association of phenotypes to genes, but possible to search by keyword and GO annotation
**WEB PORTALS**			
EuPathDB	Links to 11 other single and multi-species databases	eupathdb.org	Eukaryotic pathogen database resource for biodefense and infectious diseases on human pathogens providing an analysis tool kit to linked resources
Pathogen portal	Links to 5 database centers	www.pathogenportal.org	Focus is on pathogens as potential agents of biowarfare or bioterrorism and organisms causing (re)emerging infectious diseases (bacteria, viruses, and eukaryotes)
Phytopath	web portal	www.phytopathdb.org	Ensembl genomes browser made available as a theme group for plant pathogens. Phenotypic information is directly displayed for 32 Fungi, 14 Protists, 12 bacterial species

a virus only databases not included.

Future plans for PHI-base include the development of an online tool to allow author curation of published pathogen-host interactions from any pathogenic species. This new feature will be based on the Canto curation tool for PomBase (Rutherford et al., 2014). A refined PHI-base website will become available in 2015 to allow the display of additional manually curated information, including data on host target genes/proteins.

## Funding

This work is supported by the UK Biotechnology and Biological Sciences Research Council (BBSRC) (BB/I001077/1, BB/I000488/1, BB/K020056/1). PHI-base receives additional support from the BBSRC as a National Capability (BB/J004383/1). Funding for the open access charge was obtained from the Research Councils UK Open Access Fund.

### Conflict of interest statement

The authors declare that the research was conducted in the absence of any commercial or financial relationships that could be construed as a potential conflict of interest.
